# Duodenal Metatranscriptomics to Define Human and Microbial Functional Alterations Associated with Severe Obesity: A Pilot Study

**DOI:** 10.3390/microorganisms8111811

**Published:** 2020-11-17

**Authors:** Ilaria Granata, Carmela Nardelli, Valeria D’Argenio, Salvatore Tramontano, Debora Compare, Mario Rosario Guarracino, Gerardo Nardone, Vincenzo Pilone, Lucia Sacchetti

**Affiliations:** 1 Institute for High Performance Computing and Networking (ICAR), National Research Council (CNR), 80131 Naples, Italy; ilaria.granata@icar.cnr.it; 2 Department of Molecular Medicine and Medical Biotechnologies, University of Naples Federico II, 80131 Naples, Italy; carmela.nardelli@unina.it; 3 CEINGE Biotecnologie Avanzate S.C.a R.L., 80131 Naples, Italy; dargenio@ceinge.unina.it; 4 Task Force on Microbiome Studies, University of Naples Federico II, 80131 Naples, Italy; 5 Department of Human Sciences and Quality of Life Promotion, San Raffaele Open University, 00166 Rome, Italy; 6 Department of Medicine and Surgery, University of Salerno, 84084 Salerno, Italy; salvytra@libero.it (S.T.); vpilone@unisa.it (V.P.); 7 Department of Clinical Medicine and Surgery, University of Naples Federico II, 80131 Naples, Italy; debora.compare@unina.it (D.C.); gerardoantoniopio.nardone@unina.it (G.N.); 8 Department of Economics and Law, University of Cassino and Southern Lazio, 03043 Cassino, Italy; mario.guarracino@unicas.it

**Keywords:** metatranscriptomics, microbial-host duodenal transcriptome, human obesity, metabolism

## Abstract

Obesity is a multifactorial disorder, and the gut microbiome has been suggested to contribute to its onset. In order to better clarify the role of the microbiome in obesity, we evaluated the metatranscriptome in duodenal biopsies from a cohort of 23 adult severely obese and lean control subjects using next generation sequencing. Our aim was to provide a general picture of the duodenal metatranscriptome associated with severe obesity. We found altered expressions of human and microbial genes in the obese compared to lean subjects, with most of the gene alterations being present in the carbohydrate, protein, and lipid metabolic pathways. Defects were also present in several human genes involved in epithelial intestinal cells differentiation and function, as well as in the immunity/inflammation pathways. Moreover, the microbial taxa abundance inferred by our transcriptomic data differed in part from the data that we previously evaluated by 16S rRNA in 13/23 individuals of our cohort, particularly concerning the Firmicutes and Proteobacteria phyla abundances. In conclusion, our pilot study provides the first taxonomic and functional characterization of duodenal microbiota in severely obese subjects and lean controls. Our findings suggest that duodenal microbiome and human genes both play a role in deregulating metabolic pathways, likely affecting energy metabolism and thus contributing to the obese phenotype.

## 1. Introduction

Obesity is a pandemic disease whose prevalence has dramatically increased since the 1970s. Linear time trend forecasts indicate that, by 2030, 51% of the world population will be obese [1]. Notably, the number of severely obese individuals (body mass index (BMI) ≥ 40 kg/m^2^) has increased faster than that of obese subjects (BMI ≥ 30 kg/m^2^) [1]. Obesity is a risk factor for metabolic disorders, autoimmune diseases, and cancer [2,3,4], and dramatically impacts healthcare costs [5]. Furthermore, severely obese subjects have a much greater risk for diabetes and other diseases than mildly obese subjects and are predicted to increase healthcare costs in the future [6]. A better understanding of the pathogenic factors involved in the onset and progression of obesity may lead to interventions to reduce its spread and contain the related costs. Except for monogenic forms of obesity accounting for only 5% of severe obesity cases [7], obesity is driven by genetics, epigenetics, lifestyle, and the environment, each of which impacts the energy balance [8,9,10,11].

In the last two decades, the gut microbiota has emerged as a contributor to obesity and obesity-associated diseases [2,4,12,13,14]. The human microbiota inhabits the surfaces and niches of our organism and consists of a variety of microbes, mainly bacteria, but also fungi, viruses, and archaea, equal in number to human cells (1.3:1) [15]. In detail, the gut microbiota, and its approximately 3.3 million genes (the “microbiome”) [13], interact with the human genome and play a role in several functions, particularly in digesting food, preserving the gut mucosal barrier integrity, and priming the immune system, thus globally contributing to human health [16,17]. Thanks to the advent of next generation sequencing (NGS) techniques in the early 2000s [18], together with that of powerful tools for the bioinformatics processing of sequencing data [19,20], it has become possible to explore the gut microbiota composition in association with obesity [12,21,22,23,24]. However, given the large intra- and interindividual variability of the gut microbiota, about 20% of which is attributable to environmental factors and BMI [25], it is difficult to establish whether a microbiota is a “healthy” or “obese” one. Furthermore, metagenomically defined abundant microorganisms in the gut were found to be inactive or dormant [26]. Therefore, it is now evident that, to determine the role of the microbiome in human health or disease, its activity, as well as its composition, must be evaluated [27]. The gut microbiome has been mostly investigated in feces due to the ease of sampling [2,12,13,14], but the microbial community differs between the upper and lower intestinal tract [28]. Indeed, comparisons between the global bacterial assemblages at the phylotype level across eight different regions of the gastrointestinal tract revealed that the global bacterial structures differ significantly among regions [29]. In addition, the human small intestine is largely involved in nutrient digestion and absorption. In fact, between 85% and 95% of protein intake is digested in this tract, which is also important for simple carbohydrate metabolism and central metabolism [30,31,32]. We conducted a duodenal metatranscriptomics study in a cohort of adult severely obese (OB) and lean control subjects (CO) to identify obesity-associated alterations of both microbial and human gene expression, as well as their putative dysfunctional cross-regulation of metabolism.

## 2. Materials and Methods

### 2.1. Patients and Controls

In this study, we enrolled 12 severely obese patients (OB; 6 women and 6 men) and 11 lean control subjects (CO; 5 women and 6 men). Patients and controls were enrolled at the Surgery and Medicine Departments of the University of Salerno and of the University of Naples Federico II. Obese patients were selected among candidates for bariatric surgery and normal-weight controls were selected from among patients with gastroesophageal reflux symptoms. Inclusion criteria were an age range of 18–65 years, the filling of a weekly dietary scheme to examine specific behavioral items, and BMI ≥ 40 Kg/m^2^ or 20.0–24.9 Kg/m^2^ for obese and control subjects, respectively. Exclusion criteria were diabetes, tumors, inflammatory bowel diseases, Crohn’s disease, viral hepatitis, pharmacological treatment (i.e., antibiotics, pro- and prebiotics, antiviral or corticosteroid medications, or proton-pump inhibitors) in the last 2 months before sample collection. All patients underwent gastrointestinal endoscopy for diagnostic purposes and were enrolled after a histological examination revealed that they had a normal mucosa. The study was approved by the Ethics Committees of the Universities of Salerno and Naples (authorization n.50, 15/07/2015; amendment n. 141070, 26/11/2019 and n. 193/06, October 25, 2006; amendment n. 193/06/ESES1, October 1, 2014, respectively). All enrolled subjects gave their informed consent to participate in the study, which was carried out according to the Helsinki Declaration (2013). The clinical and anamnestic data of each subject were collected by a clinician, whereas dietary habits were self-reported and recorded by a nutritionist. Thirteen subjects (7 OB and 6 CO), belonging to our cohort, had previously been investigated by our team using 16S rRNA sequencing to characterize their duodenal microbiome composition [24].

### 2.2. Sample Collection

We collected blood samples and duodenal biopsy specimens from all enrolled individuals. The biopsy sample was taken during an upper gastrointestinal endoscopy performed for diagnostic testing, under sterile conditions to avoid contamination, as reported elsewhere [24]. Biopsies were immediately stored in a vial containing RNA Later, then cooled in dry ice and stored at −80 °C until RNA isolation for metatranscriptomics analysis. The patients’ main biochemical parameters (Table S1) were evaluated by routine assays using the ACHITECT i2000R System (Abbott Laboratories, Wiesbaden, Germany).

### 2.3. RNA Isolation and Sequencing

Total RNA was obtained from each duodenal sample. Single biopsies (10 mg/each) were pulverized to a fine powder with a standard liquid nitrogen pre-chilled mortar and pestle. This powder was transferred to a 1.5 mL tube with 500 µL of TRI Reagent solution (Ambion, Austin, TX, USA). The homogenate was vortexed vigorously for 10 min on a REAX 2000 (Heidolph, Apeldoorn, The Netherlands). After a quick spin-down, 200 µL of chloroform was added to the homogenate, vortexed for 15 s, and kept at room temperature for 5 min. The partly separated mixture was centrifuged for 15 min at 10,000 × g. The aqueous phase was transferred to a new 1.5 mL tube. RNA was purified by column precipitation, according to the mirVanaTM miRNA isolation kit (Ambion, Austin, TX, USA). At the end of the procedure, total RNA was eluted in 70 µL of 95 °C pre-warmed, nuclease-free water. Together with the duodenal samples, we processed a water sample as blank extraction control. The RNAs were quantified with the NanoDrop ND-1000 UV-Vis spectrophotometer (NanoDrop Technologies, Wilmington, DE, USA). The RNA extracts were further treated with DNase I (Qiagen, Hilden, Germany) to ensure the absence of residual DNA.

The quality and integrity of RNAs and the absence of residual DNA were assessed on the TapeStation (Agilent Technologies, Santa Clara, CA, USA) before library preparation. All samples had an RNA integrity number ranging between 6.6–8.7, which indicates good-quality samples. Starting from an RNA input/sample of 800 ng, we first depleted human and bacterial ribosomal RNAs (rRNA), followed by a cleanup using Agencourt RNAClean XP beads (Beckman Coulter, Inc., Indianapolis, IN, USA) according to standard procedures (Ribo-Zero Gold Epidemiology protocol, Illumina, San Diego, CA, USA). The quality and quantity of depleted RNA samples were checked on the TapeStation. Then, we prepared an RNA library for each depleted RNA sample using the TruSeq RNA Sample Preparation kit v2 (Illumina, Inc., San Diego, CA, USA) according to the manufacturer’s protocol. In detail, RNA samples were fragmented at 94 °C for 8 min on a thermal cycler. First-strand cDNA synthesis was performed at 25 °C for 10 min, at 42 °C for 15 min, and at 70 °C for 15 min using random hexamers and the SuperScript II Reverse Transcriptase (Thermo Fisher Scientific Inc., Waltham, MA, USA). Next, during second-strand cDNA synthesis, the RNA templates were removed, and a second replacement strand was generated by incorporation of dUTP (in place of dTTP, to keep strand information) to generate ds cDNA. AMPure XP beads (Beckman Coulter, Inc., Indianapolis, IN, USA) were used to clean up the blunt-ended cDNAs from the second-strand reaction mix. The 3′ ends of the ds cDNAs were then adenylated to facilitate adaptor ligation during the next step. After ligation of the indexing adaptors, AMPure XP beads were used to clean up the libraries a second time. Next, a PCR amplification step (15 cycles of 98 °C for 10 s, 60 °C for 30 s, and 72 °C for 30 s) was used to selectively enrich the DNA fragments that had adapter molecules on both their ends and to amplify the amount of DNA in the library. Twelve libraries (from 6 obese patients and 6 controls) were pooled to obtain 2 pools (12 samples each) for cluster generation on the cBot using the TruSeq PE Cluster Kit v3 (Illumina, Inc., San Diego, CA, USA). Finally, sequencing was performed by loading each pool in 4 lanes of a single flow cell (24 samples per run) using the TruSeq SBS kit v3, 200 cycle PE, following the manufacturer’s protocols (Illumina, HiSeq1500). The concentration of library pools for sequencing was 6 pM. All the technical controls used from RNA isolation to its sequencing, according to the manufacturers’ instructions, are detailed in the Supplementary File 1.

### 2.4. Metatranscriptomics Analysis/Processing

Metatranscriptomics sequencing data were quality controlled by using FastQC v0.11.2 [33] and subsequently deprived of adapters and low-quality reads/ends by Trimmomatic v0.38 [34].

#### 2.4.1. Human Transcriptome Analysis

Good-quality reads were aligned to the human reference genome (NCBI GRCh38) with Bowtie2 aligner v2.3.4.2 [35]. The abundances of host transcripts were quantified using the Kallisto tool v0.45.0 [36] and the counts were given as input to DESeq2 v1.24.0 [37] to determine the differential expression between the two groups under study. The genes were considered significantly different if the Logarithm of fold change ratio (Log2FC) ≥ |1| and Benjamini-Hochberg (BH) adjusted *p*-value ≤ 0.05.

#### 2.4.2. Bacterial Transcriptome Analysis

The reads that failed to map to the host genome were aligned to the phiX vector sequences using the Bowtie2 tool and, once merged, to rRNA and tRNA sequences using the SortMeRNA v2.1 tool [38]. Non-rRNA and non-tRNA sequences, theoretically corresponding to putative mRNA, were used to annotate the microbial genes and pathways. In detail, the DIAMOND program v0.9.22 [39], a superfast BLAST-based algorithm, was used to map the reads to the NCBI nonredundant bacterial protein (RefSeq_bac) and to the SEED subsystem databases (ftp://ftp.theseed.org/subsystems/) with the “BLASTX” option. One hit for each read was retained, and hits with an e-value exceeding 1e-04 or a percentage of identical matches ≤ 97% were discarded. The RefSeq hits were then aggregated by organism or function annotations using the python script “DIAMOND_analysis_counter.py” tool, while the SEED subsystem hierarchy for SEED hits was reconstructed using the python script “DIAMOND_subsystems_analysis_counter.py,” both obtained from SAMSA2 v2.2.0 pipeline [40]. In order to focus the analysis on functions linked to specific organisms of interest, we restricted the function annotations to the differentially abundant organisms, since each read processed by SAMSA software is provided by both an organism and functional annotation from the RefSeq database. This step was achieved through the python script “DIAMOND_specific_organism_retreiver.py.” We evaluated the differential gene expression using DESeq2 v1.24.0 on the abundance tables for organism and function RefSeq annotations, as well as for each of the 4 SEED subsystem level hierarchies. Terms were considered significantly different if Log2FC ≥ |1| and BH-adjusted *p*-value ≤ 0.05. Furthermore, the mRNA reads were mapped to the UniRef90 database using the HUMAnN 2.0 v2.7.8 tool [41] and the abundances obtained were combined into structured pathways from MetaCyc [42]. To identify the metabolic pathways specifically associated with obesity, we assessed the abundance of each feature in obese patients versus controls using generalized linear models in MaAsLin2 (Multivariate Association with Linear Models) [43] while controlling for age and gender covariates.

The dataset supporting the results of this article was deposited in the Sequence Read Archive (SRA) under BioProject accession code PRJNA650280.

## 3. Results

### 3.1. Severely Obese Subjects Show Significant Alterations in Blood Parameters Compared with Lean Controls

The clinical and biochemical characteristics of the study cohort are shown in Table S1. Urea (*p*-value ≤ 0.001), glucose (*p*-value ≤ 0.001), and total cholesterol (*p*-value ≤ 0.05) levels were significantly higher in blood from obese subjects than in controls, whereas albumin levels were significantly lower in obese subjects (*p*-value ≤ 0.05). The evaluation of the self-reported dietary habits highlighted important differences between OB and CO in terms of caloric intake (mean Kcal/day: 3.090 vs 1.618, respectively, *p*-value < 0.0001) and of eating habits. In fact, even if the percentages of main nutrients were similar (lipids: 28% vs 24.5%; carbohydrates: 47.7 vs 49.5; proteins: 24.09% vs 25.9%, in OB vs CO respectively), OB reported binge- and/or sweet-eating habits, 5–7 meals/day, and diets mostly containing simple and complex carbohydrates and lipids, whereas CO reported 3–4 meals/day containing preferentially complex carbohydrates and proteins.

### 3.2. The Variability of the Bacterial Transcriptome Profile Is Higher in Obese than in Control Samples

**Figure 1:**
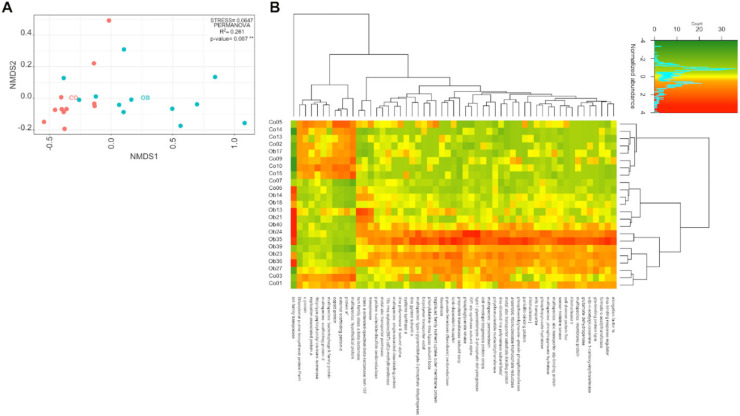
The bacterial transcriptome in Obese (OB) and Lean (CO) subjects. (**A**) Nonmetric multidimensional Scaling (NMDS) ordination of variance stabilized counts of bacterial RefSeq nonredundant proteins for CO (n = 11) and OB (n = 12) samples, compared using Bray–Curtis dissimilarity. The plot shows a higher heterogeneity in the case of obese samples and an overlapping region between the two groups, including four CO and three OB samples. The permutation analysis of variance (PERMANOVA) and corresponding R-squared and *p*-values, reported inside the plot, indicate a significant separation of the two classes. The Red and Cyan dots indicate CO and OB samples, respectively. (**B**) The heatmap-of-variance stabilized counts of bacterial RefSeq nonredundant proteins resulted in significant differences (*p* < 0.05, Log2FC > |1|) using DESeq2 differential analysis between the OB and CO groups. The Euclidean distance was calculated and the Ward’s method was used for hierarchical clustering, both for rows and columns, as shown on top and right sides of the heatmap. Two distinct clusters are observable for the two classes, indicating that most of the functions were more highly expressed in the OB group. A third cluster (at the bottom) was composed of mixed samples from the two groups.

To investigate whether the microbiota transcriptome of obese (OB) subjects differed from that of lean (CO) subjects, we aligned the reads to several sequence databases (see Materials and Methods) and extracted information at the gene, organism, subsystem, and pathway levels. The bacterial gene abundances revealed a significant difference between CO and OB samples (PERMANOVA, R^2^ = 0.261, *p*-value = 0.007), as well as a considerable intragroup variability (Figure 1A). The heterogeneity of transcription regulation looked higher in obese microbiota than in control microbiota. The differential expression analysis revealed 55 significant differentially expressed genes (DEGs) between OB and CO samples (BH adjusted *p*-value ≤ 0.05; Log2FC ≥ |1|) (Supplementary File 2). Figure 1B shows the heat map of the variance stabilized counts of the DEGs. Hierarchical clustering revealed a separation between the two groups. Indeed, all but three CO samples clustered together and showed a homogeneous expression pattern. Although the OB samples were grouped together, they had two main inner clusters, as witnessed by the hierarchical branches and the color variation inside the heat map (Figure 1B). Interestingly, most genes (45/55) were overexpressed in OB vs CO samples. In terms of metabolic function, we found that 16/45 (35.5%) of these upregulated genes were involved in nucleotide metabolism, 9/45 (20%) in carbohydrate metabolism, 9/45 (20%) in the membrane transport system, and 4/45 (8.9%) in amino acid metabolism, whereas the remaining 7/45 (15.6%) were involved in bacterial defense mechanisms. The few down-expressed genes (10/55), highlighted by the clustering performed on the rows (Figure 1B), encoded mainly scaffolding and capsid proteins (Figure 1B).

### 3.3. Prevotella and Streptococcus Are Likely the Major Contributors to Some of the Altered Microbiota Metabolic Functions in Obese Subjects

**Figure 2:**
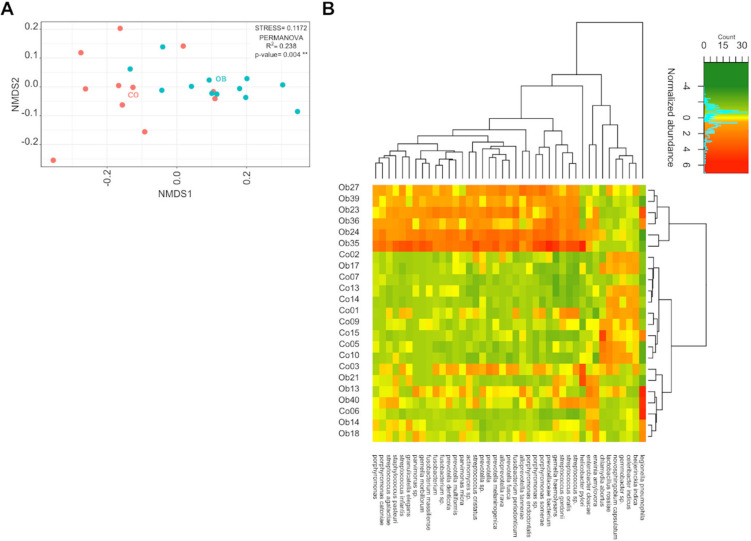
The bacterial organisms inferred from transcriptome data of Obese (OB) and Lean (CO) subjects. (**A**) Nonmetric multidimensional Scaling (NMDS) ordination of variance stabilized counts of bacterial organisms extracted from the alignment to the RefSeq nonredundant proteins database, for CO (n = 11) and OB (n = 12) samples, compared using Bray–Curtis dissimilarity. The plot shows a higher heterogeneity for both groups compared to the proteins plot (Figure 1A) and still shows an overlapping region between the two groups. The permutation analysis of variance (PERMANOVA) and corresponding R-squared and *p*-values, reported inside the plot, indicate a significant separation of the two classes. The Red and Cyan dots indicate CO and OB samples, respectively. (**B**) Heatmap of variance stabilized counts of bacterial organisms extracted from the alignment to the RefSeq nonredundant proteins database, resulted in significant differences (*p*-value < 0.05, Log2FC > |1|) using DESeq2 differential analysis between OB and CO groups. The Euclidean distance was calculated, and the Ward’s method was used for hierarchical clustering, both for rows and columns, as shown on the top and right sides of the heatmap. A distinct cluster for a subset of obese samples is observable on the top.

We evaluated taxa abundances by grouping the reads according to the organism annotations provided by the RefSeq sequences. The counts were normalized and used to perform the differential expression analysis. Figure 2A shows the nonmetric multidimensional scaling (NMDS) of samples using the normalized counts of all the organisms detected. The OB and CO cohorts were significantly separated (PERMANOVA, R^2^ = 0.238, *p*-value = 0.004). The NMDS heterogeneity was higher in the CO samples than in those of Figure 1A, which suggests that several taxa contributed to fulfil the same functions. The high variability was comparable in OB samples with that of Figure 1A, apart from the two inner groups, which were no longer distinguishable. The heat map of the significantly different bacterial organisms (DEOs) (BH adjusted *p*-value ≤ 0.05 and Log2FC ≥ |1|) is shown in Figure 2B. Interestingly, most bacterial species were more transcriptionally active in the OB than in the CO microbiomes. To investigate DEO activity, we realigned the reads to the RefSeq sequences of the organisms of interest (in this case, the DEO genera), as allowed by SAMSA2. We then analyzed the differential expression of these organism-specific genes. We obtained significant results in two cases: *Prevotella* (p_Bacteroidetes) and *Streptococcus* (p_Firmicutes). The specific functions assigned to *Prevotella* and *Streptococcus* showed significantly different results (*p*-value < 0.05) between OB and CO, as reported in Supplementary File 2. In order to verify whether the overall transcriptional activity of these two genera and their ratio were different between the two groups, we summed up all the counts matching their species obtained by grouping the reads according to the organism annotations and calculated the relative abundances at the genus (*Prevotella* and *Streptococcus*) and phylum (Bacteroidetes and Firmicutes) levels (Figure S1). *Prevotella* and *Streptococcus* abundances appeared different between OB and CO (*p*-value: 0.07 and 0.012, respectively), while the average of the abundance ratios calculated in all samples (*Streptococcus*/*Prevotella*) slightly changed between OB and CO (3.73 vs 2.44, *p*-value = 0.1). The average of the abundance ratios, summing the matches at the phylum level (Firmicutes/Bacteroidetes), was much higher in OB than in CO (5.4 vs 2.38, *p*-value = 0.06) due to the higher average abundance of Firmicutes (*p*-value = 0.0056) (Figure S1). We also calculated the abundance ratios of *Prevotella*/*Bacteroides*, the most abundant genera within Bacteroidetes and, averaging the values of samples, we found that it was higher in OB than in CO samples (5.85 vs 2.42, *p*-value = 0.019).

### 3.4. Taxa Abundance May Not Correspond to Transcriptional Activity

**Figure 3:**
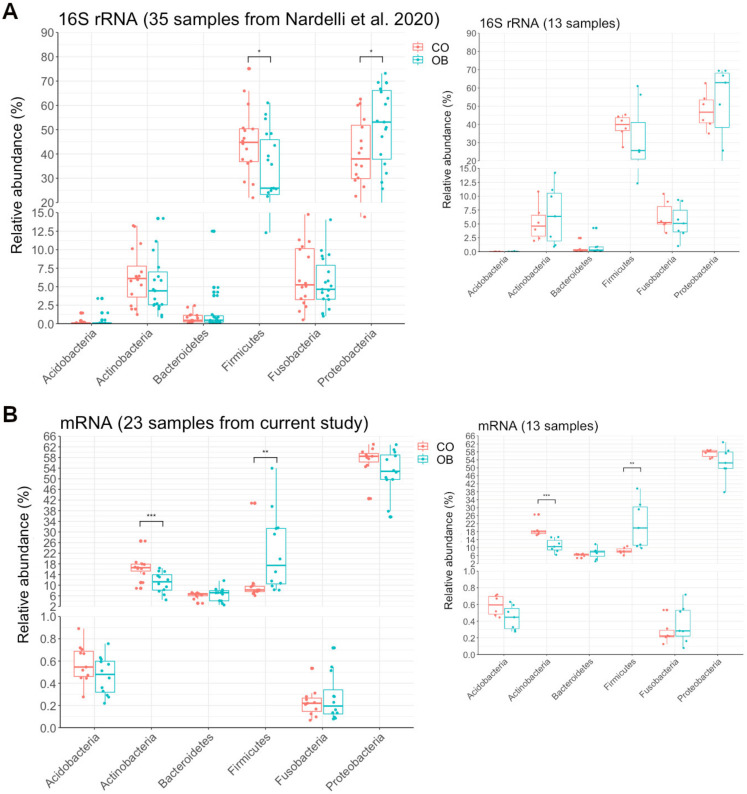
Phyla abundances in Obese (OB) and Lean (CO) subjects obtained by 16S rRNA sequencing and inferred from transcriptomic data. The boxplots show the relative abundances of phyla obtained through 16S shotgun sequencing performed in our previous study (Nardelli et al., 2020) (**A**) and inferred by the mRNA gene abundances in the current study (**B**), containing 35 and 23 samples, respectively. Among these, 13 patients (6 CO and 7 OB) were analyzed in terms of both 16S rRNA and transcriptomics and are shown by the right smaller plots. The middle line in the boxes represents the median, lower box bounds represent the first quartile, and upper box bounds represent the third quartile. Whiskers represent the 95% confidence interval of the mean. The significance of distribution differences was calculated by applying the Kruskal–Wallis test and Benjamini-Hochberg *p*-value adjustment (* *p* < 0.05; ** *p* < 0.01; *** *p* < 0.005). The Red and Cyan boxplots indicate control and obese samples, respectively.

We compared the organism abundances inferred from the alignment to the RefSeq protein sequences to those that we recently obtained using 16S rRNA sequencing in a different cohort of obese and lean control subjects, as well as in 13 samples that were common to both studies (6 CO, 7 OB) [24] (Figure 3A,B). As shown in Figure 3, the microbiome composition at phylum level, obtained from the taxonomic assignment of 16S rRNA (Figure 3A) versus that inferred from mRNA analysis performed in the present study (Figure 3B), revealed that the Firmicutes and Proteobacteria phyla were, respectively, less and more abundant in terms of 16S counts, but showed an opposite trend for the transcriptional activity. In addition, the Actinobacteria phylum was less abundant and also significantly less transcriptionally active in OB than in CO (Figure 3A,B). Similar results were obtained for both, considering the two whole cohorts of individuals and the subgroup of 13 subjects common to both experiments (see right smaller plots in A and B panels of Figure 3).

### 3.5. Carbohydrate, Amino-Acid, and Nucleotide Metabolism Are Impaired in Obese Microbiota

**Figure 4:**
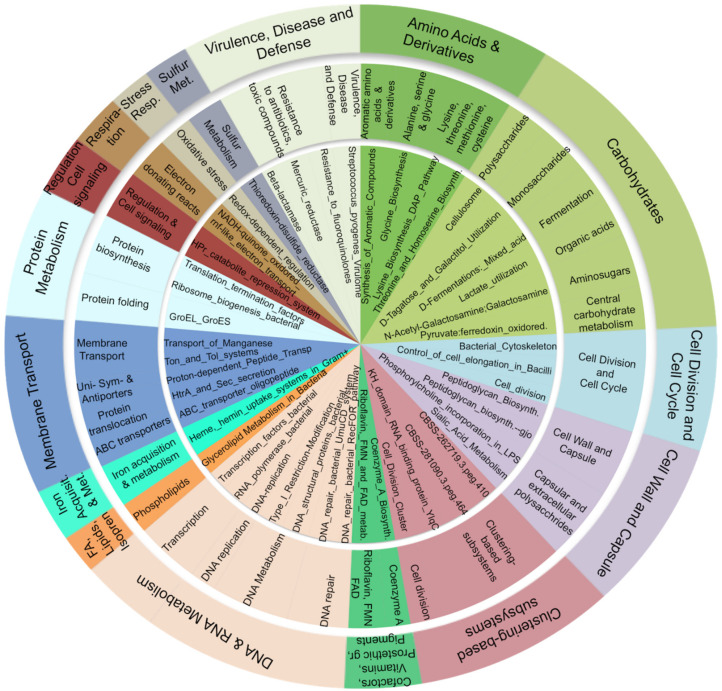
**SEED subsystem hierarchy.** The level 3 terms of SEED subsystem database (shown in the inner circle of the figure) resulted significant up-expressed genes (*p* < 0.05, Log2FC > 1) between Lean (CO, n = 11) and Obese (OB, n = 12) samples using DESeq2 differential abundance analysis. Terms were then grouped according to their belonging to higher levels 2 (middle circle) and 1 (outer circle). The slices size is proportional to the Log2FC values. The figure shows that the most altered functions concern the Amino acids, Carbohydrates, and Nucleic acids metabolism; the Clustering-based subsystem; the Virulence, Disease, and Defense mechanisms; and the Membrane transport.

**Figure 5:**
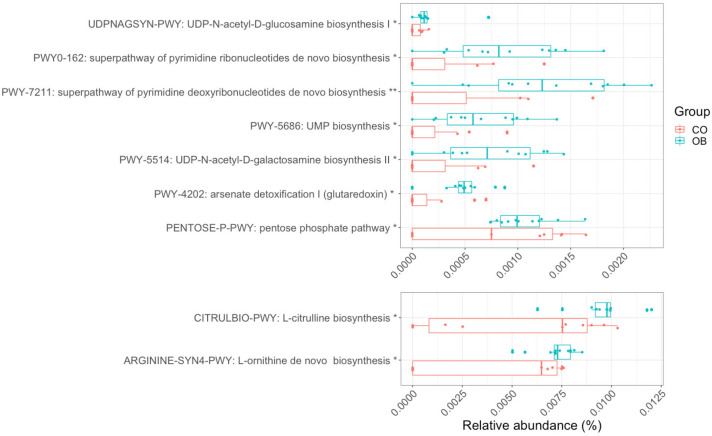
MetaCyc microbial processes associated with obesity (q < 0.1) detected using the MaAsLin2-generalized linear model. The significance was calculated after adjusting for age and gender effects. The x-axis shows the relative abundances. Boxplots were made with R function boxplot with default settings (whiskers extend 1.5-times the interquartile range). (*: 0.05 < q < 0.1: **: 0.01 < q < 0.05). Red and Cyan boxplots indicate Lean (CO, n = 11) and Obese (OB, n = 12) samples, respectively.

We exploited SEED subsystem hierarchies to group genes into functional categories and, thus, to investigate the overall activity of microbiota organisms in the OB and CO individuals. Briefly, SEED is a categorization system, which organizes functional categories of homologous genes into a hierarchy with five levels of resolution. Homologous genes are known to share the same function. The differential expression analysis was performed for each level of grouping, from 1 (the most generic) to 4 (individual functions). Level 4 (L4) and 3 (L3) differentially expressed terms are reported in Supplementary File 3. As expected, many L4 terms were redundant with the RefSeq genes. Consequently, we focused on L3, which provides an upper categorization, and the significantly upregulated terms were further grouped into level 2 and level 1 (Figure 4) to obtain a better idea of the overall functionality. The weight of the alterations and the quantity of terms involved in particular categories is reflected in the size of the slices of the pie chart, which are proportional to the Log2FC values. The figure shows that the most dominant and active functionalities in the duodenum gut microbiota of obese subjects were the metabolism of carbohydrates (16.42%), DNA and RNA (14.09%), and amino acids and derivatives (9.63%). Other upregulated terms were the Clustering-based subsystems group (terms with unknown function) (10.02%); the Virulence, Disease, and Defense mechanisms (9.03%); Membrane transport (8.92%); and Cell wall and capsule (8.57%). The microbial functional profiles were further summarized as MetaCyc pathways. In order to identify pathways specifically associated with obesity and to exclude confounding factors (covariates), such as gender and age, we performed a multivariate analysis using the generalized linear models in MaAsLin2 (Multivariate Association with Linear Models). Nine metabolic pathways were predicted to be significantly associated with obesity (Figure 5), all of which were related to the metabolism of nucleotides and amino acids.

### 3.6. Differentially Expressed Host Genes Share the Same Metabolic Pathways as Microbial Genes

We analyzed the human mRNA from the total RNAs of the duodenal niche to look for convergences between the microbiota and its host. The differential abundance analysis of human genes revealed 40 differentially expressed transcripts between OB and CO samples, of which 15 were upregulated and 25 downregulated (BH adjusted *p*-value ≤ 0.05 and Log2FC ≥ |1|) (Supplementary File 4). Based on KEGG and Gene Ontology annotations (Supplementary File 4), we grouped the genes according to their overall functional implication and found that they were mostly involved in Immunity/Inflammation/Apoptosis; the Structure and Function of Nucleic Acids; and Cellular components/functions: Membrane structure, Cytoskeleton, and Metabolism (Lipids, Carbohydrates, Proteins) (Table 1). 

**Figure 6:**
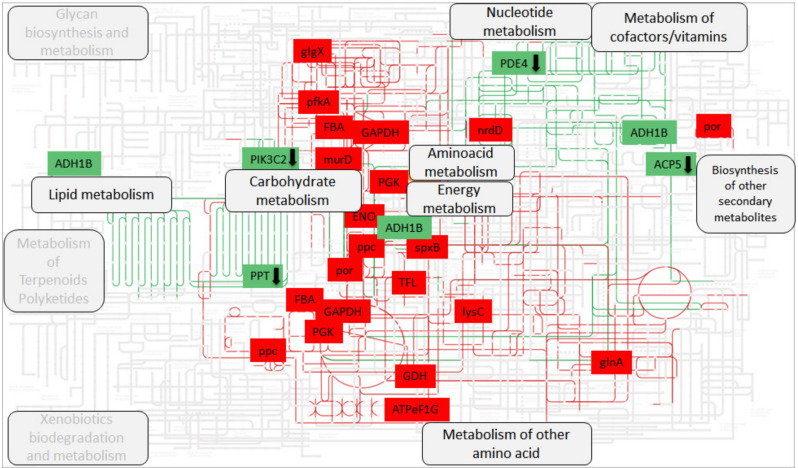
Human and microbial genes mapped into metabolism roadmap. The figure shows the metabolic pathways involving the differentially expressed genes between Obese (OB) and Lean (CO). The SEED subsystem L4 (Supplementary File 3) and human genes differentially expressed between the two groups (Table 1 and Supplementary File 4) were converted into KEGG orthologs Ids and mapped to the human metabolism using iPath3. The pathways related to the matched orthologs are highlighted to identify convergent paths between host and bacteria, colored in green and red, respectively. The genes which found one or multiple matches are also shown and highlighted by the corresponding colors. The genes reported in the figure were all up-expressed in OB vs CO, except a few which were down-expressed, indicated by an arrow in the box (n=4). Whole gene names and corresponding EC numbers are reported below in “Abbreviations” section.

Interestingly, microbial and host genes intersected at the level of Energy, Carbohydrate, and Amino Acid Metabolism (Figure 6). Other intersections occurred at the levels of Lipid and Nucleotide metabolism, in which differentially expressed host genes played a role.

## 4. Discussion

Although the gut microbiota composition is intimately associated with human health, to our knowledge, the functional activities driven by specific microbial configurations and transcription in severe obesity have not yet been elucidated. Here, we report the results of a pilot study of the human duodenal metatranscriptome in a small cohort of severely obese and lean control subjects from Southern Italy. We describe the metatranscriptomics profile associated with obesity, which includes transcripts, the contributing bacterial organisms, and the specialized pathways differentially expressed between the two groups of participants; a comparison between bacterial composition and transcriptome-related data of the human duodenal microbiome; and, finally, the putative convergence on key metabolic pathways between host and microbial transcripts, which might be associated with the obesity-associated phenotype.

### 4.1. Microbiome Composition by 16S rRNA Compared to Microbiome Inferred by Transcriptome

Recent experimental evidence has highlighted that not all the microbes detected in a human niche are equally active. Therefore, the functional activity of the microbiota should be evaluated in addition to its composition [26,27]. Using 16S rRNA gene sequencing, we recently studied the duodenal microbiome of an obese cohort of subjects from Southern Italy [24]. Here, we describe the duodenal metatranscriptome in another cohort of severely obese and lean control subjects from Southern Italy. First, we observed a high heterogeneity of the gut microbiome gene expression between and within the two groups of OB and CO subjects. Although interindividual variability is a well-known aspect of microbiota due to the many contributing aspects, this result suggests a higher microbial variability in obese subjects, which could be due to the various obesity driving factors. These results are in agreement with the high interindividual variability previously observed in the composition of human gut microbiome by 16S rRNA sequencing and mainly attributed to environmental factors and BMI [25]. Despite this, the microbial expression profiles differed significantly between OB and CO subjects. This finding indicates that a typical functional activity is associated with obesity. Furthermore, the comparison between the transcriptional activity of specific taxa inferred by transcriptome analysis, as well as their abundance previously detected by 16S rRNA gene sequencing [24], suggested that they did not show the same behavior. In detail, in the 16S rRNA analysis, Firmicutes and Proteobacteria phyla were less and more abundant in OB than in CO samples, respectively [24], and had opposite trends in the case of mRNA expression abundances. Our data support the previously observed data in agreement with species abundance and transcriptional activity in human microbiomes from fecal samples of 341 elderly men [27]. This discrepancy was not unexpected because, under steady-state conditions, transcription is a highly variable process, even among cells of the same species that may be influenced by many factors, including nutrient availability [44]. To support the nutrient influence on bacterial gene expression, D’Souza and Kost [45] experimentally demonstrated that a loss of metabolic functions was likely when the corresponding metabolites could be derived from the environment. In particular, populations of *Escherichia coli* that evolved in amino acid-replete environments rapidly lost the ability to autonomously produce several amino acids, which was beneficial when amino acids were present in the environment [45]. Essentially, we found a significantly higher functionally active Firmicutes and a higher Firmicutes/Bacteroidetes ratio in OB than in CO samples. These findings coincide with previous findings obtained in fecal samples from obese mice [46] and obese patients [47].

### 4.2. An Increased Prevotella/Bacteroides Activity Ratio Is Associated with Obesity

Among the Bacteroidetes, the *Bacteroides* and *Prevotella* genera are the most abundant, usually inhabiting the gut and oral cavity, respectively. When present in the same niche, one or the other genus predominates, acting as an antagonist [48]. They have also been suggested as biomarkers of diet and lifestyle [49]. Here, we found that the *Prevotella* genus was more active than the *Bacteroides* genus in the duodenum of obese patients compared to lean subjects. As known, *Prevotella* and *Bacteroides* characterize the two most frequently found “enterotypes” in Western and non-Western subjects, respectively, with the Prevotella-driven enterotype represented at high values in individuals consuming a carbohydrate-based diet [50]. An abundance of the *Prevotella* genus has been also associated with the Mediterranean diet [51], and an increased *Prevotella/Bacteroides* ratio has been associated with higher BMI and a higher susceptibility of obese subjects to regain weight in the case of a low dietary fiber intake and impaired glucose metabolism [52]. Our OB cohort self-reported a Mediterranean albeit hypercaloric diet (mean Kcal/day: 3.090), which contained preferentially simple and complex carbohydrates and lipids. Nevertheless, the role of diet in tipping the outcome of the competition between *Bacteroides* and *Prevotella* in the duodenum of our obese subjects remains to be ascertained.

### 4.3. The Bacterial Functional Profile in Duodenum Differs between Obese and Lean Control Subjects

Bacterial transcriptome profiling revealed differences in terms of gene expression between the severely obese and lean subjects. Below, we discuss the most relevant differences associated with obesity.

#### 4.3.1. Carbohydrate Metabolism

Using alignment to the SEED subsystem database, we found that several enzymes involved in the Embden-Meyerhoff-Parnas (EMP) pathway (namely aldolase, enolase, pyruvate kinase, phosphoglycerate kinase, pyruvate formate-lyase, and pyruvate-ferredoxin oxidoreductase) were upregulated in obese subjects. In agreement, when we grouped gene families in MetaCyc pathways, we found that the Pentose-phosphate-pathway was one of the metabolic networks associated with OB duodenal microbiomes. Bacterial carbohydrate metabolism, through glycolysis and succinate production from fumarate (first oxidative branch), as well as acetate and formate from pyruvate (second oxidative branch), are involved in the central energy and carbon metabolism of *Prevotella* spp. [53]. Despite the competitive relationship between *Prevotella* and *Bacteroides*, these two bacteria share many of the above described enzyme activities. However, unlike *Bacteroides*, *Prevotella* does not produce propionate. The latter feature could modify the abundance of some short-chain fatty acids to be used by the host’s colonocytes and could thus affect human host physiology as previously described [53]. Further, De Angelis et al. [54], using a multi-omics approach, recently studied how diet influences the functions of the human intestinal microbiome in fecal samples from volunteers with different (omnivorous, vegetarians, and vegans) dietary habits. The authors reported that phylogenetic composition was not useful in discriminating different dietary habits, but several genera were associated to the intake of specific dietary components [54]. In detail, the intestinal microbiome of individuals with vegetable-rich diets showed significant differences in relative abundances of several genes, including those involved in carbohydrate transport and metabolism [54]. In addition, higher diversity at the subgenus levels of human fecal *Prevotella* and *Bacteroides* have also previously been associated with specific dietary patterns [55]. Based on the above, we could hypothesize that the upregulation of carbohydrate metabolism found in the duodenal microbiome of our obese cohort may be diet-driven.

#### 4.3.2. Cell Wall

UDP-N-acetylgalactosamine biosynthesis is another MetaCyc pathway which was associated with OB samples. N-acetylgalactosamine is a carbohydrate molecule that, in its activated form, participates in the constitution of polymer structures, together with lipopolysaccharides, peptidoglycans, and glycans N and O, which are linked to proteins [56]. Glycosylated proteins play important roles in the cell membrane in vivo. In fact, they are often involved in cell-cell adhesion, cytoskeleton regulation, and immune recognition [56]. Lipopolysaccharide (LPS), a glycoconjugate present in the outer membrane (OM) of Gram-negative bacteria, is an important immunomodulatory molecule, contributes significantly to the structural integrity of the OM, and helps to shield the OM from antibiotics and other environmental attacks [57]. Plasma levels of LPS increased in obese animal models (ob/ob mice) as compared to lean mice in association with gut microbiota change (fewer Bacteroidetes and more Firmicutes), decreased tight junctions, and increased gut permeability [46,58]. In our study, we found the “Phosphorylcholine (ChoP) incorporation in lipopolysaccharide (LPS)” was up-expressed according to the SEED subsystem database. Interestingly, it was previously demonstrated that the attachment of the small molecule ChoP to the LPS, covering the bacterial surface, evaded immune responses during colonization in its human host [59]. We hypothesize that such mechanism could be associated with the LPS increase previously described in obesity [60].

#### 4.3.3. Defense Mechanisms: The Arsenate Detoxification Pathway

We found that the bacterial arsenate detoxification pathway was associated with obesity. Arsenic is ubiquitous in the environment and in the form of arsenate, which toxic to all living organisms. Thus, microorganisms have evolved various mechanisms to detoxify arsenate [61,62]. Interestingly, arsenate was reported to interfere with glucose, energy, and phosphate metabolism [61].

#### 4.3.4. Amino Acids and Derivatives

Gut microbiota utilizes undigested proteins, together with endogenously synthesized proteins, to generate amino acids such as arginine, which is converted via citrulline into L-ornithine, a precursor for polyamine production whose increased levels negatively affect gut health [63]. Interestingly, among the significantly up-expressed MetaCyc pathways in our obese cohort were those of L-ornithine and L-citrulline biosynthesis, which are both alternative arginine amino acid metabolic pathways that can lead to proinflammatory or immunoregulatory effects [63].

#### 4.3.5. Nucleic Acids

The de novo pyrimidine biosynthesis pathway predominates during intensive growth in mammals and most other organisms [64]. The latter pathway enables these organisms to synthesize nucleotides from small metabolites. The de novo pyrimidine biosynthesis pathway is also essential for the proliferation of many pathogens [64]. We found the de novo pyrimidine biosynthesis pathway was up-expressed in our cohort. Thus, we may speculate that it could contribute to obesity-associated dysbiosis.

### 4.4. The Human Tanscriptional Profile in the Duodenum Differs between Obese and Lean Subjects

Most of the human transcripts in the duodenum of our OB patients were down-expressed compared to lean subjects. In particular, the down-expression of ErbB3 and of CSF1R genes, observed in our obese cohort, was previously described to alter intestinal cells differentiation [65,66]. Indeed, ErbB3 is expressed in the small intestine, and its signaling restricts the number of Paneth cells (PCs). This effect requires PI3K signaling [65], which also was down-expressed in our obese subjects. Accordingly, the intestinal epithelium of ErbB3 (-/-) mice was sensitized to inflammation and the PC expansion was altered, thereby decreasing the release of antimicrobial peptides [65]. Regarding CSF1R, this gene is expressed in macrophages, which are associated with the crypt epithelium and involved in the maintenance of the intestinal stem cell niche and PC differentiation [66]. As known, Paneth cells release antimicrobial lysozyme and α-defensins, together with other factors essential for the maintenance of intestinal stem cells [67]. Globally, defects in PCs or in their production of antimicrobial factors can result in intestinal dysbiosis [66]. Based on the above assumptions, we may hypothesize that the down-expression of ErbB3 and of CSF1R genes, observed in the duodenum of our obese subjects, could be associated with gut epithelial dysfunction and dysbiosis. Another down-expressed gene in our obese group was AnxA1. The latter participates in cell differentiation, membrane transport, motility, signal transduction, inflammation, and apoptosis [68]. Interestingly, the relationship between AnxA1, obesity, and obese-associated metabolic diseases has been well documented due to the role of AnxA1 in the treatment of type-2 diabetes [69,70,71,72]. In fact, AnxA1-null mice fed a high-fat diet were more obese and had larger adipocytes than wild-type mice fed a high-fat diet, whereas recombinant human AnxA1 treatment reduced body weight, fat mass, and liver steatosis [72]. Intestinal epithelial integrity and cell-to-cell adhesion are essential to ensure gut function and homeostasis. In fact, tight junctions control paracellular permeability (gate functions) [73]. The other deregulated transcripts in our obese cohort were IQGAP1, AnxA2, calmodulin, and WDR1. Interestingly, all of them could be involved putatively in intestinal cell membrane composition, gate function, and inflammatory bowel diseases [68]. In detail, the IQ motif containing GTPase-activating protein1 (IQGAP1), a member of a family of scaffolding proteins, is involved in the differential recruitment of claudins to nascent tight junctions [74]. Among the 300 interacting proteins documented for IQGAP1, AnxA2 and calmodulin were both up-expressed in duodenum of obese subjects. In particular, calmodulin competes for binding to IQGAP1 both in vitro and in a normal cellular milieu [75]. Further, WD-repeat protein1 (WDR1), also known as “actin-interacting protein 1,” plays a significant role in actin cytoskeletal remodeling by promoting the assembly of apical cell-cell junctions [76]. Lastly, the down-expression of Lamin in OB duodenum could likely contribute to the deregulation of gene expression associated with obesity. In fact, Lamins A/C are nuclear proteins that polymerize to form the nuclear lamina, which is a filamentous network located just below the inner nuclear membrane that assists in maintaining nuclear membrane integrity and controlling gene expression [77]. Accordingly, defective A-type lamins have been previously described in conditions such as regional adiposity, type-2 diabetes, and aging [78].

Globally, the human transcriptome profile of duodenum found in our obese cohort could likely be responsible for the dysfunction of the intestinal epithelium composition, intestinal permeability function, and inflammation, all of which may be associated with the obese-associated dysbiosis and may alter key metabolic pathways.

### 4.5. Putative Convergence between Host and Microbial Transcriptomes

Intriguingly, among the human obese-associated transcripts, the up-expressed Alcohol dehydrogenase 1B (ADH1B) and the down-expressed Palmitoyl-protein thioesterase 1 (PPT1) and Phoshatidylinositol-4-phospate 3-kinase subunit beta (PIK3C2B) were all involved in the metabolic pathways to which bacteria also contribute, and may be potentially be associated with the obese phenotype. In fact, the expression of ADH1B was found altered in the adipose tissue of obese subjects and was associated with BMI and waist circumference [79]. A deficiency of PPT1 activity and altered cholesterol metabolism have been reported in brain tissue in association with local neuroinflammation [80], as well as in Sertoli cells in association with male subfertility, abnormal lysosome accumulation, and increased cholesterol levels [81]. In agreement with the above data, the down-expression of PPT1 observed in our OB subjects was associated with significantly increased serum cholesterol levels. Finally, PIK3C2B, which we found to be down-expressed, is an enzyme ubiquitously expressed in human tissues and a controller of endosome trafficking, whose inactivation alters insulin receptor trafficking [82].

Our pilot study presents some limits. First, we studied a small cohort. However, all subjects were from the Campania Region of Southern Italy and self-reported similar dietary habits, thereby likely minimizing dietary and environmental differences. Second, duodenal samples were taken when the subjects were in a fasting state, and intestinal function might vary over time (i.e., after meal assumption) [44]. Finally, the described obese-associated transcriptomic profile requires further metabolomics and/or proteomics validation. To the best of our knowledge, this is the first report on duodenal metatranscriptomic profiles in obese subjects. If our findings are confirmed in larger cohorts than ours, from various geographical areas and different lifestyles, we could hypothesize therapeutic strategies attempts (i.e., prebiotics, probiotics, or antibiotics) aimed at producing a desired change in microbial composition and/or function that favourably impacts the host metabolism, hopefully reducing obesity and the risk of obese-associated diseases. Furthermore, the evaluation of the influence of single nutrients on the obese-associated microbiota and microbiota-host interaction may provide further insights on diet-based treatments for the management of obese subjects.

## 5. Conclusions

Globally, we provided a global picture of duodenal transcriptome perturbations associated with severe obesity. Duodenum is a gastrointestinal region whose bacterial assemblage differs from that of other more frequently studied regions and scarcely investigated in obesity, notwithstanding its importance for the nutrients’ digestion. We found that microbial composition and functional activity only partially coincided. Indeed, the carbohydrate and lipid metabolic pathways were among those most actively deregulated in the duodenum of obese subjects. Defects in human genes involved in epithelial intestinal cell differentiation, permeability function, and inflammation may also contribute to the obesity-associated gut dysbiosis.

## Figures and Tables

**Table 1:** Significantly up-expressed and down-expressed human genes in Obese (OB) vs Lean (CO) and their putative role (based on KEGG and GOTERM databases).

Transcripts		Localization	Log2FC	Adjusted *p*-Value	Putative Role *
BCL2	Apoptosis regulator	Mitochondrial membrane	−1.82	0.004	**Immunity/Inflammation/ Apoptosis**
TNFRSF13B	TNF receptor superfamily member 13B	Plasma membrane	−1.44	0.031	
CSF1R	Colony stimulating factor 1 receptor	Membrane	−1.62	0.037	
ACP5	Acid phosphatase 5, tartrate resistant	Lysosome	−1.06	0.027	
IGLL5	Immunoglobulin lambda like polypeptide 5	Extracellular localization	−1.75	0.027	
ANXA1	Annexin 1	Different cellular localization	−2.12	0.039	
ERBB3	Erb-b2 receptor tyrosine kinase 3	Plasma membrane	−24.56	<0.0001	
NGEF	Neuronal guanine nucleotide exchange factor	Membrane	3.66	0.025	
P4HB	Prolyl 4-hydroxylase subunit beta	Cytoplasm	21.74	<0.0001	
PRMT5	Protein arginine methyltransferase 5	Nucleus and cytoplasm	−7.43	0.0001	**Nucleic acids structure and functions**
TSEN34	tRNA splicing endonuclease subunit 34	Nucleus	−5.8	0.031	
EIF3CL	Eukaryotic translation initiation factor 3 subunit C-like	Cytoplasm	21.82	<0.0001	
ELF2	E74 like ETS transcription factor 2	Nucleus and cytoplasm	6.94	0.008	
WBP2	WW domain binding protein 2	Nucleus and cytoplasm	2.88	0.0022	
ZNF195	Zinc finger protein 195	Nucleus	3.21	0.033	
SRRT	Serrate, RNA effector molecule	Nucleus	6.18	<0.0001	
PDE4A	Phosphodiesterase 4	Cytoplasm	−1.22	0.015	
ANXA2	Annexin 2	Different cellular localization	1.69	0.012	**Cellular components/functions: membrane structure, cytoskeleton**
IQGAP1	IQ motif containing GTPase activating protein 1	Plasma membrane	−1.73	0.008	
WDR1	WD repeat domain 1	Different cellular localization	−24.6	<0.0001	
ANXA1	Annexin 1	Nucleus	−2.12	0.039	
BANF1	Barrier to autointegration factor 1	Different cellular localization	−1.15	0.041	
CDR1	Cerebellar degeneration related protein 1	Nucleus	−2.53	0.012	
FAM192A	Family with sequence similarity 192 member A	Plasma membrane	−5.59	0.014	
PARD3	Par-3 family cell polarity regulator	Cytoplasm and membrane	−8.32	0.023	
PLEKHB1	Pleckstrin homology domain containing B1	Plasma membrane	−1.23	0.012	
RGMA	Repulsive guidance molecule family member a	Cytoplasm	−4.68	0.037	
CLASP1	Cytoplasmic linker associated protein 1	Plasma membrane	3.80	0.014	
CD82	CD82 molecule	Membrane	5.78	0.041	
DAG1	Dystroglycan 1	Membrane	4.47	0.009	
ILDR1	Immunoglobulin like domain containing receptor 1	Membrane	6.24	0.027	
LMNA	Lamin A/C	Nucleus	−9.63	0.028	
CASC4	Cancer susceptibility candidate 4	Membrane	7.62	<0.0001	
MME	membrane metalloendopeptidase	Membrane	20.61 (P)	<0.0001	**Metabolism: lipid (L), carbohydrates (C), protein (P)**
PPT1	palmitoyl-protein thioesterase 1	Lysosome	−1.37 (L)	0.014	
MGEA5	Meningioma expressed antigen 5 (hyaluronidase)	Nucleus	−24.08 (C)	<0.0001	
RAP1GDS1	Rap1 GTPase-GDP dissociation stimulator 1	Cytoplasm	−4.02 (L)	0.045	
LMNA	Lamin A/C	Nucleus	−9.63 (L+C)	0.028	
PGA3	Pepsinogen 3, group I	Different cellular localization	−5.92 (P)	0.031	
CALM2	Calmodulin 2	Different cellular localization	10.91 (L+C)	<0.0001	
PIK3C2B	Phosphatidylinositol-4-phosphate 3-kinase catalytic subunit type 2 beta	Cytosol	−7.39 (L)	0.027	
ADH1B	Alcohol dehydrogenase 1B	Different cellular localization	3.41 (L+C)	0.005	
ANXA2	Annexin 2	Different cellular localization	1.69 (L+C)	0.012	
BCL2	Apoptosis regulator	Mitochondrial membrane	−1.82 (L)	0.004	

* Based on KEGG and GEOTERM database.

## Supplementary Materials

The following are available online at https://www.mdpi.com/2076-2607/8/11/1811/s1, Figure S1. Barplots showing the relative abundances of *Prevotella*, *Streptococcus*, *Bacteroides* genera and corresponding Bacteroidetes and Firmicutes phyla. The barplot on the right shows the values of OB and CO samples of the ratios *Firmicutes*/*Bacteroidetes*, *Prevotella*/*Bacteroides* and *Streptococcus*/*Prevotella*. The mean ± SE is indicated for each bar. The significance of distribution differences has been calculated applying the Kruskal-Wallis test (* *p* < 0.05; ** *p* < 0.01); Supplementary File 1. Additional methodological details regarding samples collection, RNA isolation with technical controls and sequencing procedures; Supplementary File 2. Excel File containing the significant RefSeq functions (sheet 1), organisms (sheet 2) and functions specifically associated to *Prevotella* and *Streptococcus* genera (sheet 3). For each term, the corresponding log2FC (OBvsCO) and adjusted *p*-value are also reported; Supplementary File 3. Excel File containing differentially expressed L3 (sheet1) and L4 (sheet2) SEED subsystem terms and their correspondent upper levels. Log2FC values and BH adjusted *p*-values are also reported. The different colors refer to L1 categorization; Supplementary File 4. Excel File containing up- (sheet1) and down- (sheet2) regulated host transcripts between OB and CO. For each Ensembl transcript id are reported the following annotations: the Ensembl gene Id, the Gene symbol, the Log2FC value, the BH adjusted *p*-value, the Gene name, the GO Biological Processes, the GO Molecular Functions and the KEGG pathways.; Table S1. Clinical and biochemical characteristics of lean (CO) and obese (OB) subjects.

## Abbreviations

GAPDH	NAD-dependent glyceraldehyde-3-phosphate dehydrogenase (EC 1.2.1.12)
spxB	Pyruvate oxidase (EC 1.2.3.3)
GDH	NADP-specific glutamate dehydrogenase (EC 1.4.1.4)
PFL	Pyruvate formate-lyase (EC 2.3.1.54)
pfkA	6-phosphofructokinase (EC 2.7.1.11)
PGK	Phosphoglycerate kinase (EC 2.7.2.3)
lysC	Aspartokinase (EC 2.7.2.4)
ppc	Phosphoenolpyruvate carboxylase (EC 4.1.1.31)
FBA	Fructose-bisphosphate aldolase class II (EC 4.1.2.13)
ENO	Enolase (EC 4.2.1.11)
glnA	Glutamine synthetase type I (EC 6.3.1.2)
murD	UDP-N-acetylmuramoylalanine--D-glutamate ligase (EC 6.3.2.9)
ATPeF1G	ATP synthase gamma chain (EC 3.6.3.14)
glgX	Glycogen debranching enzyme (EC 3.2.1.196)
por	Pyruvate-flavodoxin oxidoreductase (EC 1.2.7.1)
nrdD	Ribonucleotide reductase of class III (anaerobic), large subunit (EC 1.17.4.2)
PIK3C2B	phosphatidylinositol-4-phosphate 3-kinase catalytic subunit type 2 beta (EC 2.7.1.154)
PPT	palmitoyl-protein thioesterase 1 (EC 3.1.2.22)
PDE4	phosphodiesterase 4 (EC 3.1.4.53)
ADH1B	alcohol dehydrogenase 1B (class I), beta polypeptide (ADH1B) (EC 1.1.1.1)
ACP5	acid phosphatase 5, tartrate resistant (EC 3.1.3.2)
