# Supplementary material for: Duodenal Metatranscriptomics to Define Human and Microbial Functional Alterations Associated with Severe Obesity: A Pilot Study
Source: Microorganisms. 2020 Nov 17;8(11):1811. doi: 10.3390/microorganisms8111811 (PMC7698607; doi:10.3390/microorganisms8111811)
Supplement: Supplementary file 1 [file microorganisms-08-01811-s001.zip › Suppl final/Supplementary Table S1.docx]

**Table S1.** Clinical and biochemical characteristics of lean (CO) and obese (OB) subjects.

|  | **CO (n=11)** | | **OB (n=12)** | |
| --- | --- | --- | --- | --- |
|  | **Mean** | **SD** | **Mean** | **SD** |
| Age, years | 54.9 | 17.5 | 41.6 | 9.9 |
| BMI, kg/m^2 *^ | 23 | 1.1 | 46.1 | 4.15 |
| Systolic blood pressure, mmHg | nd | nd | 138.08 | 7.47 |
| Diastolic blood pressure, mmHg | nd | nd | 86.5^#^ | 80–90^#^ |
| Heart rate, beats/min | nd | nd | 81,9 | 8,07 |
| Iron, μg/dL | 104.7 | 20.38 | 89.7 | 34.7 |
| Urea, mmol/L ^*^ | 16.5^#^ | 13–17.75^#^ | 34.9 | 9.1 |
| Glucose, mmol/L ^*^ | 4.2 | 0.69 | 5.3 | 0.4 |
| Insulin, mIU/L | 6.4^#^ | 4.6–9.07^#^ | 10.1 | 2.18 |
| Creatinin, μmol/L | 0.89 | 0.2 | 0.95 | 0,18 |
| Total proteins, g/L | 6.95 | 0.46 | 7.39 | 0.77 |
| Albumin, g/L ^**^ | 4.56 | 0.51 | 4.22 | 0.5 |
| Uric acid, mmol/L | 4.04 | 0.87 | 5.4^#^ | 5.2–6.78^#^ |
| Total bilirubin, μmol/L | 0.68 | 0.25 | 0.65^#^ | 0.47–0.77^#^ |
| Total cholesterol, mmol/L ^**^ | 4.4 | 1 | 5.7 | 1.2 |
| Triglycerides, mmol/L | 1.18 | 0.5 | 1.5 | 0.6 |
| HDL-cholesterol, mmol/L | 1.3 | 0.3 | 1.3 | 0.17 |
| AST, U/L | 21.8 | 8.74 | 21.5^#^ | 19.25–30.0^#^ |
| ALT, U/L | 38.3 | 10.3 | 28.9 | 13.09 |
| ALP, U/L | 62^#^ | 56.0–66.0^#^ | 51^#^ | 50.7–62.2^#^ |
| GGT, U/L | 22.5 | 8.42 | 18^#^ | 14.5–25.2^#^ |
| Amylase, U/L | 62.7 | 22.8 | 46.5 | 17.03 |

* p < 0.001; ^**^ p < 0.05. # median value and 1st and 3rd quartile were reported for nonparametric distributions.
