# Supplementary figures and images for: Duodenal Metatranscriptomics to Define Human and Microbial Functional Alterations Associated with Severe Obesity: A Pilot Study
Source: Microorganisms. 2020 Nov 17;8(11):1811. doi: 10.3390/microorganisms8111811 (PMC7698607; doi:10.3390/microorganisms8111811)

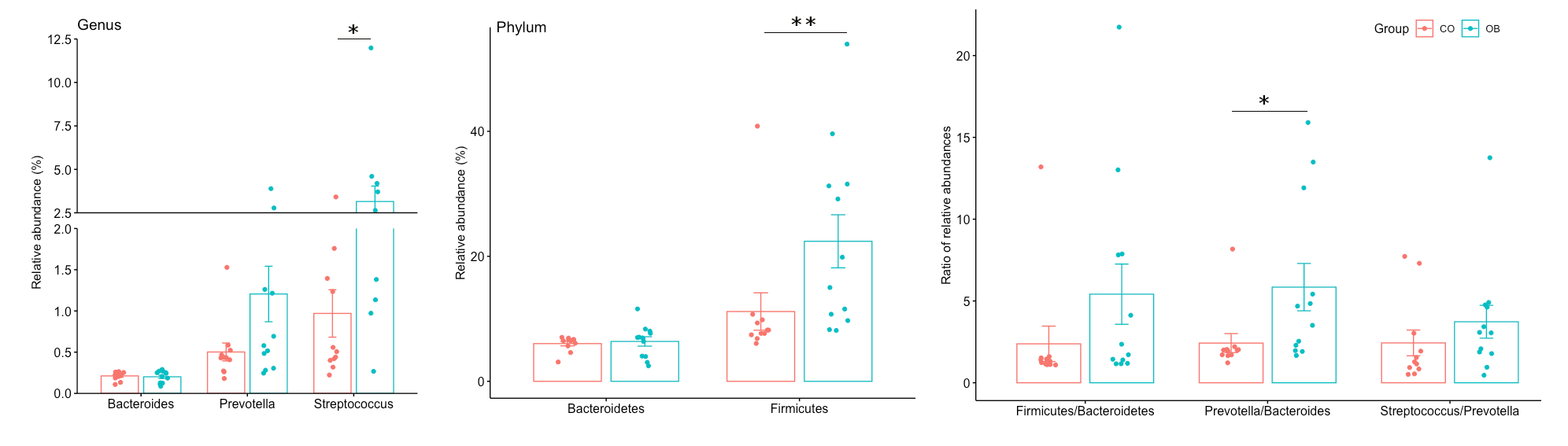

Supplement: Supplementary file 1 [file microorganisms-08-01811-s001.zip › Suppl final/Supplementary_Figure_1.png]
