# Supplementary material for: Duodenal Metatranscriptomics to Define Human and Microbial Functional Alterations Associated with Severe Obesity: A Pilot Study
Source: Microorganisms. 2020 Nov 17;8(11):1811. doi: 10.3390/microorganisms8111811 (PMC7698607; doi:10.3390/microorganisms8111811)
Supplement: Supplementary file 1 [file microorganisms-08-01811-s001.zip › Suppl final/Supplementary file 1.doc]

Supplementary File 1

Supplemental Materials and Methods

**Sample collection and processing**

**Sampling**

Collection of duodenal biopsies by endoscopy: in this step, including an appropriate negative control is not easy task, particularly when the studied samples are faeces or biopsies. In fact, sampling the air or container with a different instrument could not be a true negative control since no material should be available for DNA/RNA extraction. Consequently, for this step we did not have negative controls, but our goal was to minimize technical variations, as reported below.

In order to reduce contamination risk, all biopsies were performed using standardized procedures. In particular, all endoscopes were reprocessed before use according to high-level disinfection protocols including manual washing, automated endoscope washer reprocessing and adequate drying/storage after rinsing (Multisociety guideline on reprocessing flexible gastrointestinal endoscopes: Gastrointestinal Endoscopy. 2011;73:1075-1084) defined as the destruction of all vegetative microorganisms, mycobacteria, small or nonlipid viruses, medium or lipid viruses, fungal spores and some, but not all, bacterial spores. All samples were taken with sterile biopsy forceps and the endoscopist wore sterile gloves and a mask. The samples used for the study were invariably taken before the other samples used for histological examination. Oral washing was performed for all patients before endoscopic study of enrolled patients: oral contamination can be considered the smallest possible, although unavoidable. After endoscopic biopsy, specimen was carefully extracted, without oral or gastroesophageal passage or contact. All single-use containers were used for all biopsies. All specimens were immediately placed in a sterile tube on dry ice and transferred to a –80°C freezer within 15 min and until DNA/RNA extraction. The same procedure was performed in the two different clinical centers which contributed equally to the enrolment of both obese and controls groups. We retain that this sampling procedure was effective in minimizing risk of contaminations, both external and internal.

**Sample processing**

All our samples (both from control and obese subjects) were processed concurrently in the same laboratory at Center of advanced Biotechnology CEINGE scarl, center that has implemented and maintains a quality management system which complies with the standard UNI EN ISO 9001:2015 for the diagnostic activities among which those performed using next generation sequencing.

In this framework, the same researcher, reagents, and equipment were used to process all the samples of the present study. Personnel wore protective clothing and equipment to cover all exposed surfaces (i.e., disposable gloves, lab-coat, face-mask) and processed samples in controlled environment, as recently suggested (Eisenhofer, R. et al. Cell Press Reviews. Trends in Microbiology, 2019;27,105-17).

**RNA isolation**

The RNA extraction of all samples was performed in a pre-PCR designed room under a laminar-flow hood to protect laboratory staff, samples and experiments from contaminations. Pre-PCR work was physically isolated from the post-PCR work. We extracted RNA from all samples by using the same TRI Reagent solution and mirVanaTM miRNA isolation kit and according manufacter’s instructions.

RNA extraction kits and other laboratory reagents could be source of contamination. A negative control of extraction was performed processing a tube containing only water and the reagents of kit.

RNA quantity was evaluated with the NanoDrop® ND-1000 UV-Vis spectrophotometer (NanoDrop Technologies, Wilmington, DE, USA). The RNA extracts were further treated with DNase I (Qiagen, Hilden, Germany) to ensure the absence of residual DNA. The quality and integrity of RNAs and the absence of residual DNA were assessed on the TapeStation (Agilent Technologies, Santa Clara, CA, USA) before library preparation. All samples had an RNA integrity number ranging between 6.6–8.7, which indicates good quality samples. The negative control of RNA extraction was confirmed as negative.

To exclude a genomic DNA contamination in our RNA samples we performed an RNA TapeStation assay. Considering the indications reported in the Application Compendium - Applications for DNA, RNA, and Protein Analysis of Agilent 2100 Bioanalyzer System - Agilent 4200 TapeStation System (Figure 1, upper panel), we verified the RNA TapeStation profiles obtained in our samples and observed that in all samples was not detected the genomic DNA contamination. We showed as example of the RNA profile obtained in one of our samples (CO13) (Figure 1, lower panel).

**Figure 1.** RNA analysis by TapeStation System. The upper panel reports an image from the Application Compendium - Applications for DNA, RNA, and Protein Analysis of Agilent 2100 Bioanalyzer System - Agilent 4200 TapeStation System. This image shows a RNA profile before and after DNAase treatment. The asterix (*) in the picture indicates a genomic contamination. The lower panel below shows a RNA profile obtained in one of our samples (CO-13), reported as example. All our RNA samples showed a similar profile that we do not show for simplicity.

**Figure 2.** Screenshot from TapeStation analysis of both gel image (on the left) and sample info (on the right) of some RNA samples included in the present study.

Figure 2 shows the screenshot of both gel image (on the left) and sample info (on the right) relative to some of our RNA samples. Among those present in the gel showed in Figure 2 on the left, we choose to include in the present study the RNAs of 5/12 obese patients (OB14, OB23, OB24, OB35, OB39) and 8/11 controls (CO09, CO13, CO14, CO1, CO2, CO3, CO6, CO7). The other RNAs here showed were excluded because they did not meet the quality requirements. The other samples included in the present paper were not here showed, for simplicity, but their RNA profiles were similar to those showed in these images.

**RNA sequencing**

The RNA libraries were carried out in a PCR designed room under a PCR hood with HEPA-filtered vertical laminar flow to ensure the contamination control and PCR process repeatability. An integrated UV lamp enabled rapid decontamination of the work zone between experiments and prevents cross-contamination. Nuclease-Free Water (Invitrogen) was used for reagent mix preparation.

Starting from an RNA input/sample of 800 ng, we first depleted human and bacterial ribosomal RNAs (rRNA) followed by a cleanup using Agencourt RNAClean XP beads (Beckman Coulter) according to standard procedures (Ribo-Zero Gold Epidemiology protocol, Illumina, San Diego, CA, USA). The quality and quantity of depleted RNA samples were checked on the TapeStation (**Figure 3**).

**Figure 3.** Depleted human and bacterial ribosomal RNA (Co13 sample as example).

Then, we prepared an RNA library for each depleted RNA sample using the TruSeq RNA Sample Preparation kit v2 (Illumina, Inc., San Diego, CA, USA) according to the manufacturer’s protocol. In detail, RNA samples were fragmented at 94°C for 8 minutes on a thermal cycler. First-strand cDNA synthesis was performed at 25°C for 10 minutes, at 42°C for 15 minutes, and at 70°C for 15 minutes using random hexamers and the SuperScript II Reverse Transcriptase (Thermo Fisher Scientific Inc., Waltham, MA, USA). Next, during second-strand cDNA synthesis, the RNA templates were removed and a second replacement strand was generated by incorporation of dUTP (in place of dTTP, to keep strand information) to generate ds cDNA. AMPure XP beads (Beckman Coulter, Inc., Indianapolis, IN, USA) were used to clean up the blunt-ended cDNAs from the second-strand reaction mix. The 3’ ends of the ds cDNAs were then adenylated to facilitate adaptor ligation during the next step. After ligation of indexing adaptors, AMPure XP beads were used to clean up the libraries a second time. Next, a PCR amplification step (15 cycles of 98°C for 10 seconds, 60°C for 30 seconds and 72°C for 30 seconds) was used to selectively enrich the DNA fragments that had adapter molecules on both their ends and to amplify the amount of DNA in the library. The quality and quantity of libreries were checked on the TapeStation (**Figure 4**).

**Figure 4.** Example of library (Co13 sample as example).

Twelve libraries (from 6 obese patients and from 6 controls) were pooled to obtain 2 pools (12 samples each) for cluster generation on the cBot using the TruSeq PE Cluster Kit v3 (Illumina). Finally, sequencing was performed by loading each pool in 4 lanes of a single flow cell (24 samples per run) using the TruSeq SBS kit v3, 200 cycle PE, following the manufacturer’s protocols (Illumina, HiSeq1500). The concentration of library pools for sequencing was 6 pM.
